# Cerebral Salt-Wasting Syndrome in Severe Brain Trauma: A Case Report and Review of the Literature

**DOI:** 10.7759/cureus.81504

**Published:** 2025-03-31

**Authors:** Adrian Baracan, Ana-Maria Baracan, Mara Moldovan

**Affiliations:** 1 Faculty of Medicine, Transilvania University of Brasov, Brasov, ROU; 2 Anesthesia and Intensive Care, "Regina Maria" Military Emergency Clinical Hospital, Brasov, ROU; 3 Internal Medicine, Brasov County Clinical Emergency Hospital, Brasov, ROU; 4 Anesthesia and Intensive Care, Brasov County Clinical Emergency Hospital, Brasov, ROU

**Keywords:** cerebral salt wasting syndrome, fractional excretion, hyponatremia, phosphate, syndrome of inappropiate antidiuretic hormone secretion, uric acid

## Abstract

Sodium disorders are common in critically ill patients with brain injury. The severity of symptoms and long-term adverse neurological outcomes are related to the degree of sodium abnormality, the time the disorder has developed, and the treatment. After severe brain trauma, hyponatremia occurs most frequently associated with cerebral salt-wasting syndrome (CSWS) or the syndrome of inappropriate antidiuretic hormone (ADH) secretion (SIADH). We present the case of a patient with severe brain trauma who developed CSWS, followed by a systematic review of the literature. This case report includes the clinical and laboratory criteria used for the differential diagnosis between CSWS and SIADH.

## Introduction

Hyponatremia is a serum sodium concentration of <135 mmol/L [1]. The classification of hyponatremia may be based on biochemical severity when the ion-specific electrode measures serum sodium and can be mild (Na+ values between 130 and 135 mmol/L), moderate (Na+ values between 125 and 129 mmol/L), and profound (Na+ below 125 mmol/L). One can also take into account the time of development (acute when it occurred less than 48 hours and chronic if the disorder debuted more than 48 hours) and symptoms (moderately and severely symptomatic) [1,2].

Neurological symptoms usually occur when the serum sodium concentration falls below 120 mmol/L [3]. Clinical early recognition of the entity causing hyponatremia is almost impossible, as symptoms are nonspecific [3]. Initial symptoms may include headache, lethargy, nausea, muscle cramps, weakness, and can progress to confusion, hallucinations, psychosis, and dysarthria. If cerebral edema is severe, patients may present with seizures, coma, respiratory arrest, hemiplegia, and even death [3].

Hyponatremia is common after brain injury, usually developing between two and seven days after the injury [4]. Cerebral salt-wasting syndrome (CSWS), a state of hypovolemic hyponatremia, complicates traumatic brain injury (TBI), subarachnoid hemorrhage, and neurosurgery [5].

In the intensive care setting, CSWS must be differentiated from the syndrome of inappropriate antidiuretic hormone (ADH) secretion, a euvolemic hyponatremia. Implementing the wrong treatment has been shown to increase mortality, increase the length of stay in the intensive care unit, and increase the cost of hospitalization [3].

Although CSWS was first described in 1950, its pathophysiologic mechanism has not been well-understood until recently. First, the central nervous system (CNS) injury disrupts the autonomic nervous system stimulation of proximal tubular sodium and urate reabsorption, resulting in natriuresis and increased diuresis; second, brain injury might increase the secretion of human atrial natruretic peptide (hANP) and brain natriuretic peptide (BNP), which inhibits sodium reabsorption, thus resulting in increased natriuresis and diuresis [3,4,6,7].

The differential diagnosis between CSWS and SIADH is based on clinical and laboratory investigations: body weight, plasma volume, fluid balance, serum sodium concentration, serum osmolality, urinary sodium concentration, urinary osmolality, fractional excretion of uric acid (FEUA), and FE of phosphate (FEP) [4,8].

Even though CSWS is a hypovolemic disorder while SIADH is not, evaluation of extracellular fluid can be complex. Evaluating volemia is an important clinical characteristic to help differentiate between the two syndromes. CSWS is a hypovolemic hyponatremia, induced by increased natriuresis and diuresis. On the other hand, SIADH is an euvolemic hyponatremia, characterized by lack of edema and normal blood pressure [3,4,6-8]. Further, renal FEUA and FEP can be considered a diagnostic guide [3].

The FE of a substance is determined by measuring the levels of that substance, as well as creatinine, in both blood and urine. To rearrange the formula for FE, it can be expressed as the ratio of the urine concentration of the substance to its plasma concentration, divided by the ratio of the urine concentration of creatinine to its plasma concentration [9,10].

Considering the FE of uric acid, one must know that both CSWS and SIADH have a high FE (over 10%) at the beginning. Only after hyponatremia's correction does FEUA in SIADH lower, but in CSWS, it remains high [3].

Fractional phosphate excretion is an essential parameter, and there is limited research in diagnosing CSWS from the first detection of hyponatremia in TBI. FEP is normal (<10%) in SIADH, and increased (> 20%) in CSWS, being a criterion for distinguishing these pathologic conditions [3]. We present a case of hyponatremia in a TBI patient that was early diagnosed as CSWS by using FEUA and FEP along with the other hemodynamic and biochemical criteria.

## Case presentation

A 30-year-old female smoker with no other medical history was admitted to the intensive care unit with the diagnosis of multiple trauma produced by a motor vehicle crash. She presented with severe head trauma: Glasgow Coma scale (GCS) score of 5 points (E2V1M2), bilateral basal pulmonary contusion, and right humerus fracture.

**Figure 1:**
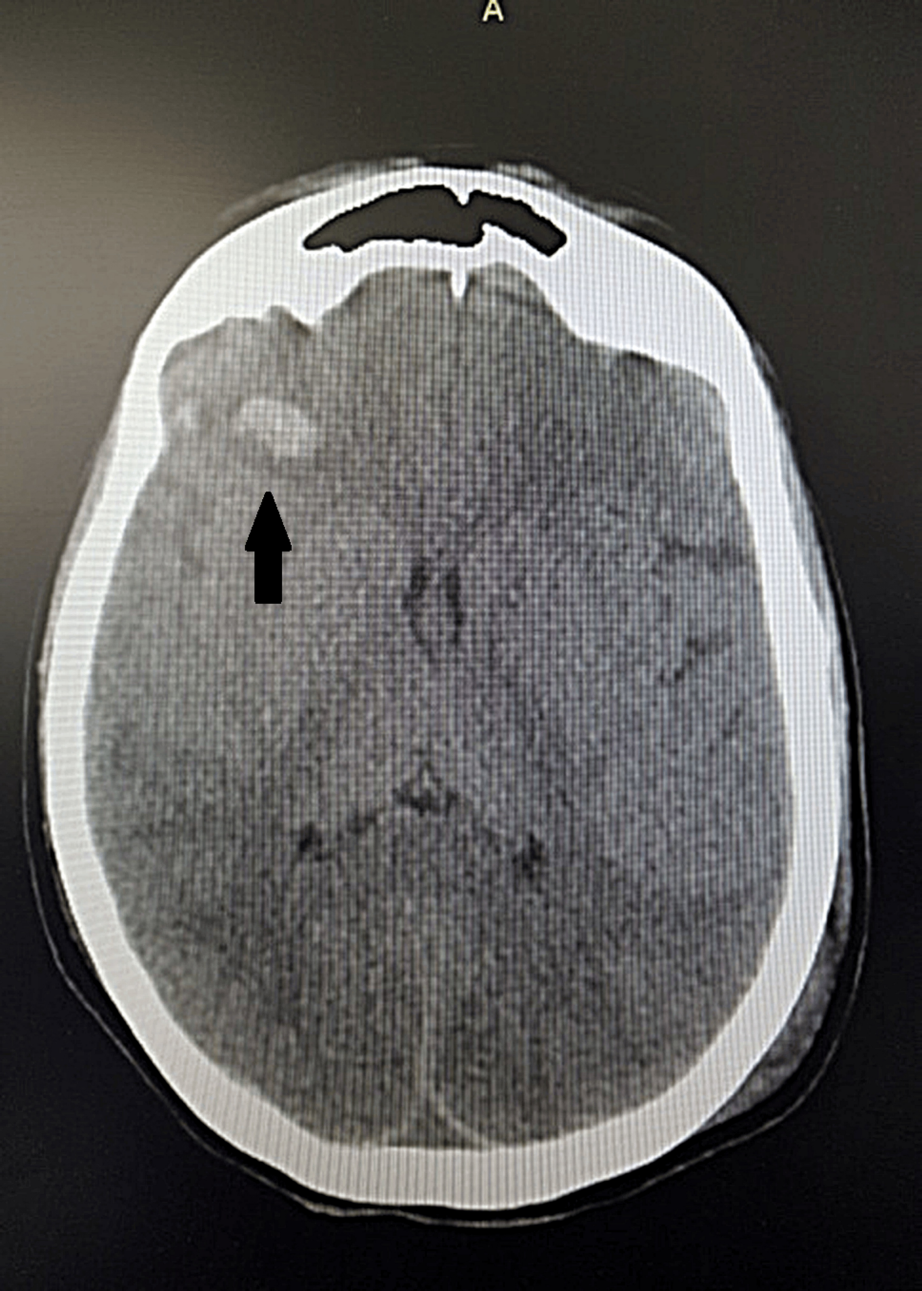
Computer tomographic axial cross-section of the brain, showing a right frontal hemorrhagic contusion

**Figure 2:**
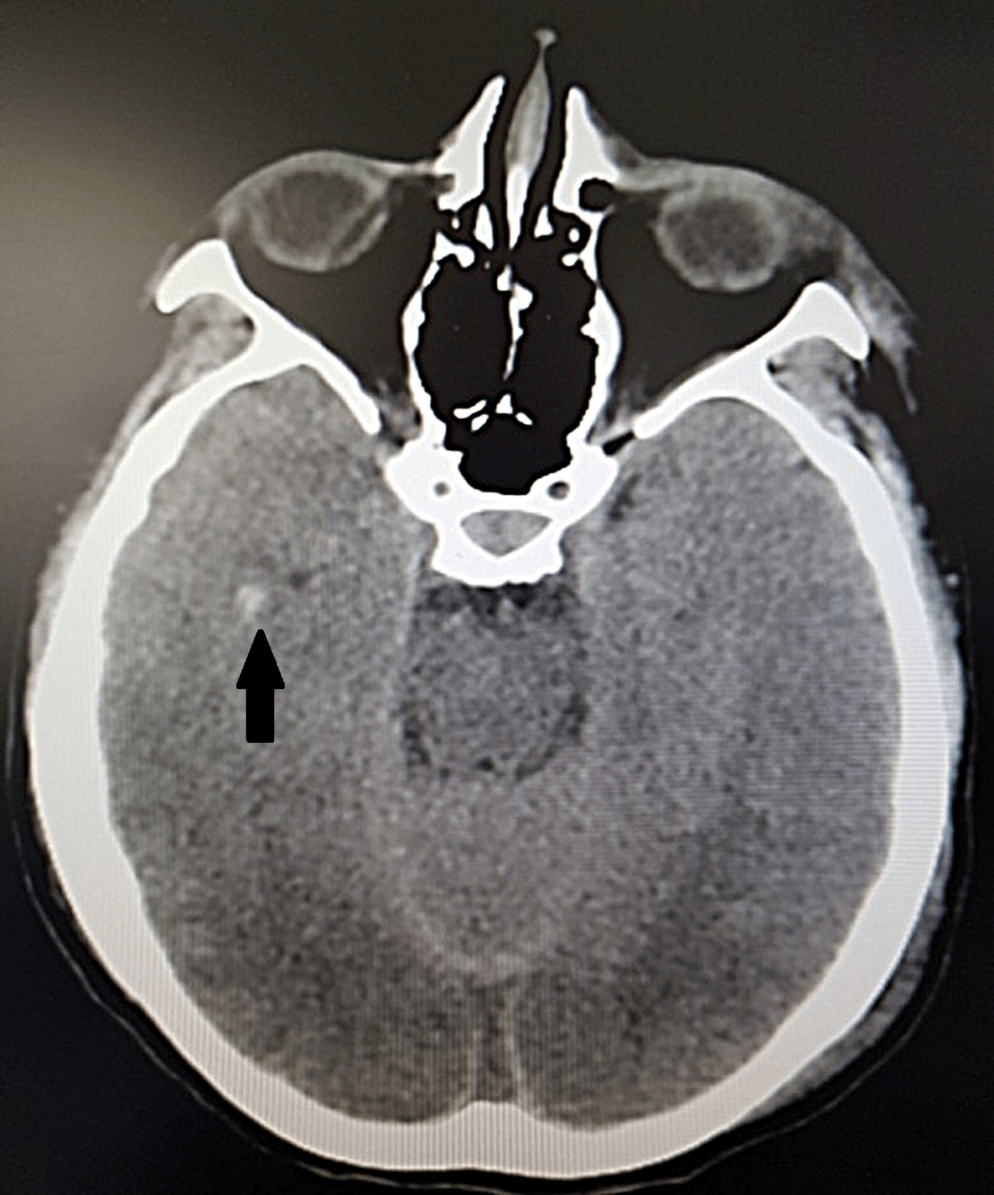
Computer tomographic axial cross-section of the brain showing a right temporal bleeding

The CT head examination revealed severe TBI: right frontal hemorrhagic contusion, right temporal bleeding, subarachnoid hemorrhage in the cerebellar tentorium, and intergiral at the vertex level, low symmetrical cerebral ventricles (Figures 1, 2).

She was intubated for airway protection. A right subclavian central venous catheter and an arterial line on the right radial artery were placed. The transducer for the arterial line was positioned at the level of the external auditory meatus, allowing continuous monitoring of the mean arterial pressure (MAP). A urinary catheter and a feeding tube were also placed. The patient required emergency surgery for internal fixation of the fractured humerus and postoperatively was admitted to the ICU and remained orotracheally intubated and mechanically ventilated.

Biologically, on admission, the patient showed normal leukocyte count, normal renal and hepatic function, moderate anemia, and mild rhabdomyolysis. Through her ICU stay, her hepatic and renal function remained normal. 

The patient was positioned in the ICU with her thorax elevated at a 30-degree angle. She received convulsion prophylaxis, stress ulcer prophylaxis, and deep vein thrombosis (DVT) prophylaxis. Normocapnia and normoglycemia were maintained throughout her care. Cerebral depletion was managed with the administration of a 20% mannitol solution.

From the second day of admission, the patient became hyponatremic and polyuric (with a urinary output of 4,23 mL/kg/h). Serum sodium was 136.8 mg/dL on admission and decreased to a minimum of 118 mg/dL on day 3. Further, the patient became hemodynamically unstable, with a positive passive leg raise test, thus requiring fluid resuscitation with normal saline to increase preload and the addition of noradrenaline to maintain adequate cerebral perfusion pressure.

Based on the patient's hypovolemia despite adequate fluid resuscitation, CSWS became a presumptive diagnosis; thus, the decision was made to determine FEAU and FEP. Table 1 indicates the values of plasma and urinary osmolality, plasma and urine sodium, and excretion fractions of sodium, uric acid, and phosphate. FEAU was high (58%), FEP was high (31%), and the CSWS diagnosis was formulated by correlating the other clinical and biochemical data.

**Table 1:** Serum and urinary parameters of the patient. Differential diagnosis between CSWS and SIADH CSWS: cerebral salt-wasting syndrome; SIADH: syndrome of inappropriate antidiuretic hormone; FE: fractional excretion

Parameter	Patient value	Reference value	SIADH	CSWS
Body weight	62 kg	-	↑	↓
Plasma volume	1845 mL	50-55 mL/kg	↑	↓
Fluid balance	Negative	-	Positive	Negative
Serum Na (mmol/L)	118	135 to 145	↓	↓
Urinary Na (mmol/L)	115	Less than 20	↑	↑
SOsm (mosm/L)	250	285 to 295	↓	↑
UOsm (mosm/L)	325	50-1500	↑	↑
FE Na %	1,82%	-		
FE urea %	40%	-		
FE uric acid %	58%	5% to 10%	↑	↑
FE phosphate %	31%	Less than 20 %	↓	↑

Table 2 presents the evolution of acid-base parameters, sodemia, and diuresis. Hyponatremia was corrected using a 3% NaCl infusion, with rates calculated according to the Androgue-Madias formula. Care was taken to adjust infusion rates so that they did not increase by more than 4-6 mEq in 24 hours and thus to avoid pontine myelinolysis. Corrected serum sodium values are shown in Table 2. Normal saline was also administered to replace losses from diuresis.

**Table 2:** Arterial blood acid-base parameters and diuresis HCO_3_-: bicarbonate; BE: base excess; Na^+^: sodium

Day/parameter	1	2	3	4	5	6	7	Reference range
pH	7.45	7.41	7.43	7.42	7.43	7.45	7.45	7.35 to 7.45
HCO_3_^-^ (mmol/L)	25	22	22	26	25	25	23	22 to 28
BE (mmol/L)	+1.9	-1.2	-2	+1.3	+1.2	+1.3	+0.6	-2 to +2
Lactate (mmol/L)	1.56	0.96	0.9	0.76	0.81	1.82	1.2	Less than 1
Na^+^ (mmol/L)	136.8	125	118	124	129	134	138	135 to 145
Diuresis (mL/day)	2100	6300	9300	7400	8700	3800	3300	0.5 to 1.5 mL/kg/day

**Figure 3:**
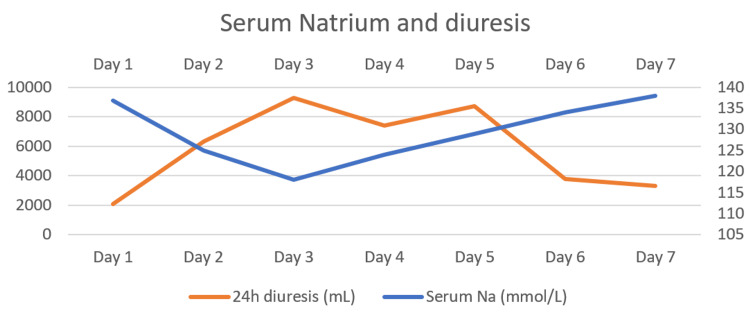
Serum sodium and diuresis trend

Figure 3 shows the dynamics of plasma sodium values and urinary output during her first week in the ICU. On day 3, urinary output was highest at 6.25 mL/kg/h (9300 mL in a 24-hour period), and it correlates with the lowest plasma sodium value (118 mEq/L). After that, plasmatic sodium levels started to respond to treatment, with a rise varying between 5 and 6 mEq per day.

On the sixth day, sodium values neared the lower limit of the reference interval (134 mmol/L), and polyuria remitted. The 3% NaCl infusion was stopped, and the patient continued to be perfused with normal saline to maintain a normal fluid balance.

The patient's evolution was favorable. After ten days of intensive care, she was discharged to the ward without the need for supplementary sodium other than the daily requirements for her weight.

## Discussion

This case is clinically relevant because it confirms the importance of applying biochemical criteria for the correct differential diagnosis of hyponatremia in traumatic brain injury. It is essential to correctly differentiate between CSWS and SIADH, as some studies showed that fluid restriction in patients with hyponatremia not associated with SIADH can lead to shock and permanent brain damage [11].

Another study [11] showed that urine Na excretion and volume measurement are significant, as both these parameters are significantly higher in CSWS than in SIADH. Moreover, patients with SIADH have a clinically normal blood volume status (blood, plasma, RBC volumes), comparable with normonatremic patients, and not comparable with CSWS, where there is a clinically observed fluid deficit [12]. But what of patients with concomitant acute comorbidities associated with hypovolemia, for which evaluation of fluid status is not that easy? What are the rest of the criteria for diagnosing CSWS?

Even though CSWS was first described 75 years ago, to our knowledge, there are no official diagnostic criteria, although some have been suggested. A narrative review published in 2018 [3] concludes that using FEUA and the FEP is safe and easily differentiates between SIADH and CSW.

We conducted a systematic review to find other similar case reports. We searched three databases, Scopus, MEDLINE, and Web of Science, from their inception until March 1, 2025. The search strategy included ("cerebral salt wasting syndrome" OR CSWS) AND (trauma OR TBI or "Traumatic brain injury" OR "cerebral hemorrhage" OR injury OR brain).

The inclusion criteria were as follows: case reports on adults with traumatic brain injury who developed CSWS, articles that specify the diagnostic method, and studies in English

The exclusion criteria were as follows: articles other than case reports (randomised controlled trials, book chapters, reviews, letters to the editor, observational studies), studies in a language other than English, animal studies, studies on the pediatric population, and CSWS of etiologies other than traumatic.

**Figure 4:**
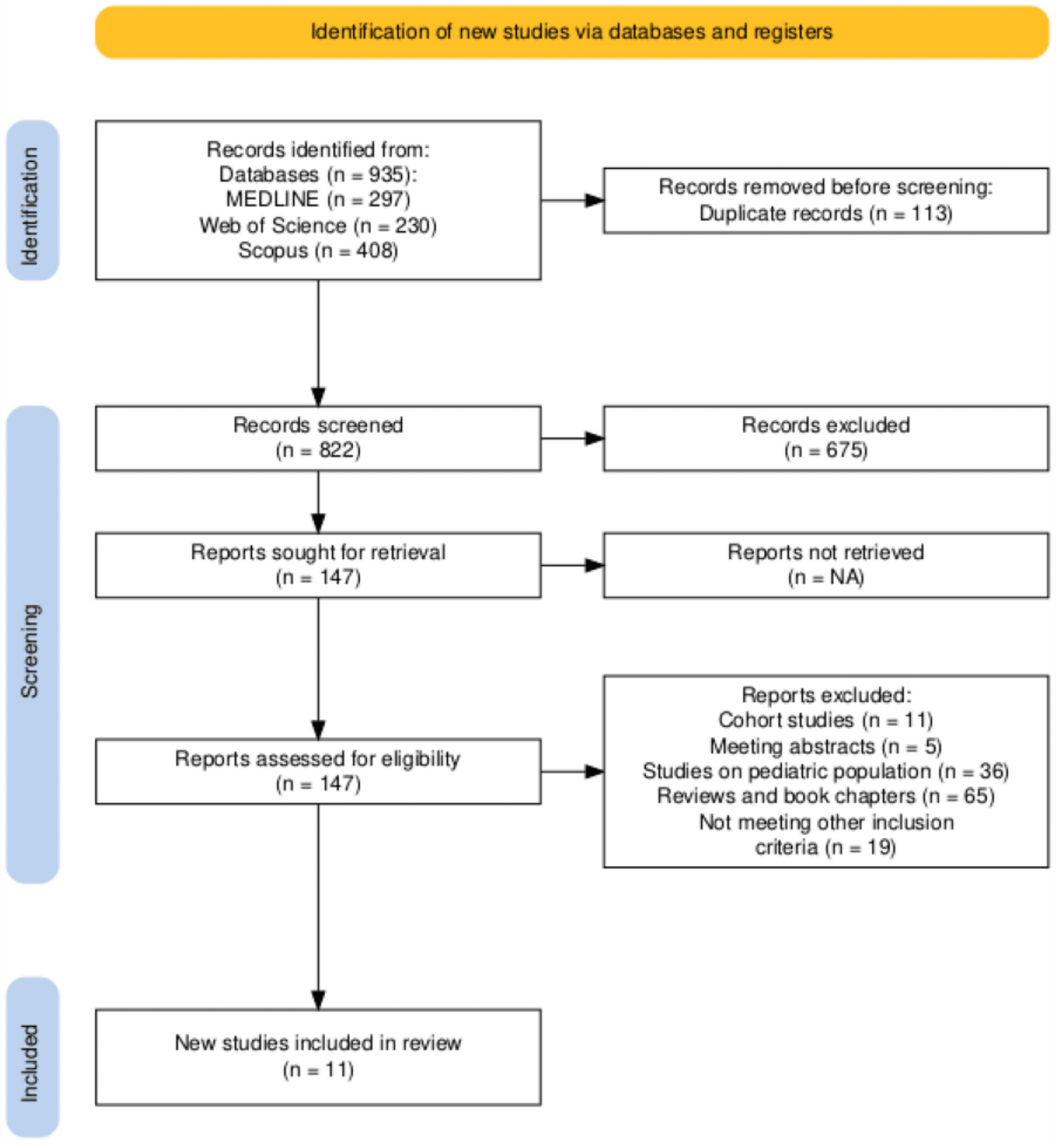
PRISMA flow diagram for the systematic search PRISMA: Preferred Reporting Items for Systematic Reviews and Meta-Analyses

Figure 4 illustrates the Preferred Reporting Items for Systematic Reviews and Meta-Analyses (PRISMA) flow diagram, showing the process of identification, screening, and finally including relevant articles in the systematic review. Table 4 summarizes the search results, showing the 11 studies reviewed.

**Table 3:** Case studies on CSWS in TBI

Nr. Crt.	Study, year	Age of the patient	Type of craniocerebral trauma
1.	Nakajima et al., 2016 [7]	60	Traumatic subarachnoid hemorrhage
2.	Lu et al., 2008 [13]	52	Large right holo hemispheric subdural hematoma with 8-mm midline shift, right frontal contusions, intraventricular hemorrhage, right uncal herniation.
		48	Left frontal intraparenchymal hemorrhage with left frontal subdural hematoma and no midline shift.
3.	Csipak et al., 2016 [14]	23	Comminuted fracture of the nasal bone and fracture of the internal wall of the maxillary sinus.
		19	Minor head trauma without neurological or neurosurgical problems.
4.	Fukuoka et al., 2017 [15]	34	Thin subcutaneous hematoma on the right forehead. No skull fracture, intracranial hemorrhage, or brain contusion on computer tomography scan.
5.	Shen et al., 2017 [16]	53	Subarachnoid hemorrhage, diffuse axonal injury.
		65	Extradural hematoma, subarachnoid hemorrhage, brain contusion, diffuse brain swelling.
		31	Extradural hematoma, subarachnoid hemorrhage, bifrontal contusion.
		41	Diffuse axonal injury, subarachnoid hemorrhage, diffuse brain swelling.
6.	Hoai et al., 2020 [17]	44	Right temporal hemorrhage 1.4x1.5 cm, scattered subarachnoid hemorrhages in the right hemisphere with compression of the right ventricle. Rupture of the right jaw, nose fracture, and nasal septum.
7.	Junhai et al., 2020 [18]	69	Bilateral subdural effusion
8.	Mohamed et al., 2021 [6]	48	Subarachnoid hemorrhage
9.	Nagamine, 2021 [19]	70	Small subarachnoid hemorrhage in the right inferior temporal sulcus, the left superior frontal sulcus, and a small left subdural hemorrhage.
10.	Sciacovelli et al., 2024 [20]	81	Minor head injury with no fractures or hemorrhages on a computer tomography scan.
11.	Rojas-Urrea et al., 2024 [21]	25	Multiple bullet shrapnel in the temporal lobe and left parietal lobe, compromise of the greater wing of the sphenoid, sphenoidal sinus, and left maxillary sinus, and subarachnoid hemorrhage.

A total of 11 case reports were found. None of the studies made the diagnosis of CSWS by determining FEP, and only one determined FEUA [7].

Symptomatic hypovolemia guided the diagnosis in most of the studies [6,7,14,15,17,18,20,21]. Two studies out of eleven (18.18%) diagnosed CSWS after excluding SIADH when hyponatremia did not respond to fluid restriction [7,19].

All the patients but those from one study [13] had a positive outcome and were discharged from the hospital with the resolution of CSWS. In four studies [7,16,18,19], there was a need for the administration of a corticoid concomitant with sodium repletion.

Intriguingly, in two studies [14,15], a diagnosis of CSWs was made in patients with minor head trauma. In two other studies [17,19], CSWS was associated with mild brain injury (with small hemorrhages on CT scan), without focal neurological signs. Even though some studies show that CSWS is associated with more severe TBI [22], there is a possibility that this syndrome also appears in minor TBI; thus, it is suggested to monitor sodium levels and fluid balance in TBI patients with a GCS score of <13 [22].

Another finding was that these two syndromes can become interchangeable. A study documented the concurrence of CSWS with SIADH [16], while another [7] showed that a patient with traumatic SAH developed CSWS, and after resolving, he further developed SIADH. Hence, there may be some limitations in diagnosing CSWS based on hypovolemic status only.

Our study involved a CSWS that occurred following TBI. The particularity of the case was that it was diagnosed early by the aid of FEUA and FEP and treated with correction of sodium deficit, with a favorable outcome.

## Conclusions

The diagnosis of SIADH and CSWS must be based on clinical and biochemical criteria. Plasma volume depletion and polyuria are characteristic of CSWS. The most essential laboratory parameters for the differential diagnosis are serum and urinary osmolality, FEUA, and FEP. Because the therapy of these two pathologic states is opposed, a precise diagnosis of the type of hyponatremia in patients with acute cerebral injuries is mandatory from the beginning to reduce the morbidity and mortality.
